# Supplementary material for: Voltage-controlled topological spin textures in the monolayer limit
Source: Nat Commun. 2026 Feb 19;17:2923. doi: 10.1038/s41467-026-69800-7 (PMC13031274; doi:10.1038/s41467-026-69800-7)
Supplement: Supplementary file 1 — Supplementary Information [file 41467_2026_69800_MOESM1_ESM.pdf]

Supplementary Materials for

Voltage-controlled topological spin textures in the monolayer limit

Yangliu Wu[1,2,§], Bo Peng[1,2,§] ✉, Zhaozhuo Zeng[3,§], Chendi Yang[4], Haipeng Lu[1,2], Peiheng Zhou[1,2], Jianliang Xie[1,2], Difei Liang[1,2], Linbo Zhang[1,2], Peng Yan[3] ✉, Haizhong Guo[5,6] ✉, Renchao Che[4] ✉, and Longjiang Deng[1,2] ✉

[1]National Engineering Research Center of Electromagnetic Radiation Control Materials, School of Electronic Science and Engineering, University of Electronic Science and Technology of China, Chengdu 611731, China

[2]Key Laboratory of Multi Spectral Absorbing Materials and Structures of Ministry of Education, School of Electronic Science and Engineering, University of Electronic Science and Technology of China, Chengdu 611731, China

[3]School of Physics and State Key Laboratory of Electronic Thin Films and Integrated Devices, University of Electronic Science and Technology of China, Chengdu, 610054, China

[4]Laboratory of Advanced Materials, Department of Materials Science, Collaborative Innovation Center of Chemistry for Energy Materials(iChEM), Fudan University, Shanghai 200433, China

[5]School of Physics, Zhengzhou University, Zhengzhou 450052, P. R. China

[6]Institute of Quantum Materials and Physics, Henan Academy of Sciences, Zhengzhou 450046, China

[§]These authors contributed equally: Yangliu Wu, Bo Peng, Zhaozhuo Zeng

✉To whom correspondence should be addressed. Email address: bo_peng@uestc.edu.cn; denglj@uestc.edu.cn; rcche@fudan.edu.cn; yan@uestc.edu.cn; hguo@zzu.edu.cn

The PDF file includes:
Supplementary Text
Supplementary Note 1-3
(Supplementary Fig. 1 to 14)

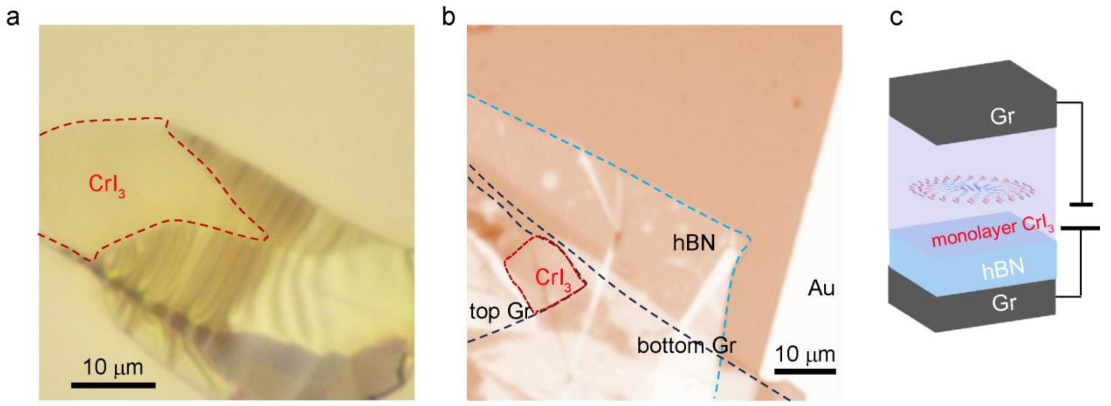

Supplementary Fig. 1 | Optical microscope images of monolayer (1L) CrI_3 and its van der Waals heterostructure device 1. a Optical microscope image of CrI_3 nanosheets exfoliated from bulk CrI_3 and adhered to the PDMS surface, and the region enclosed by the red dashed line represents the atomic-thick CrI_3 area. **b** False-color optical micrograph of the van der Waals heterostructure device, with the junction region represented by the area enclosed by a red dashed line. **c** Schematic of a CrI_3 monolayer device prepared by layer-by-layer restacking.

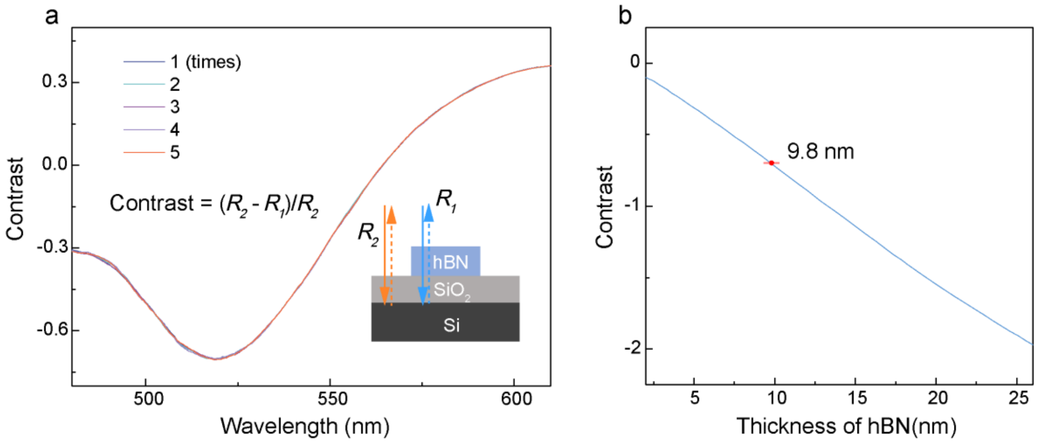

Supplementary Fig. 2 | The thickness of the insulating dielectric hBN in the device. **a** The optical contrast of hBN on the SiO_2/Si substrate as a function of the light wavelength. The inset provides the testing method and calculation formula for the contrast, where R_2 and R_1 correspond to the reflectance spectra on the SiO_2/Si substrate and on the substrate with hBN, respectively. **b** The light blue solid line illustrates the linear dependence of optical contrast at 516 nm on the thickness of the hBN nanosheet. The red dot in the figure marks the dielectric hBN thickness in the device, approximately 9.8 nm. The error bars represent mean ± standard error of the mean.

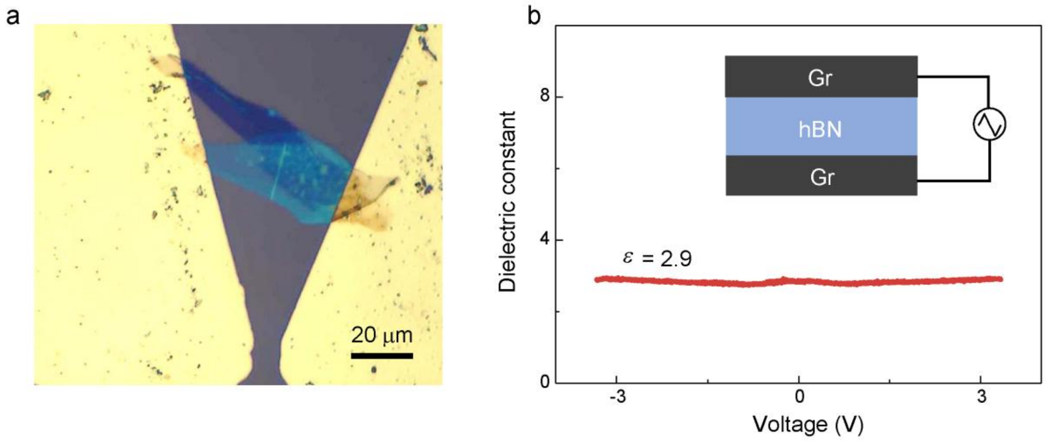

Supplementary Fig. 3 | The dielectric constant of the hBN in the device. To examine the dielectric properties of pristine hBN, a pristine hBN nanosheet was selected to prepare a graphene/hBN/graphene capacitor for dielectric constant testing. **a** Optical image of the graphene/hBN/graphene capacitor device. **b** Dielectric constant as a function of voltage, measured using a ferroelectric tester.

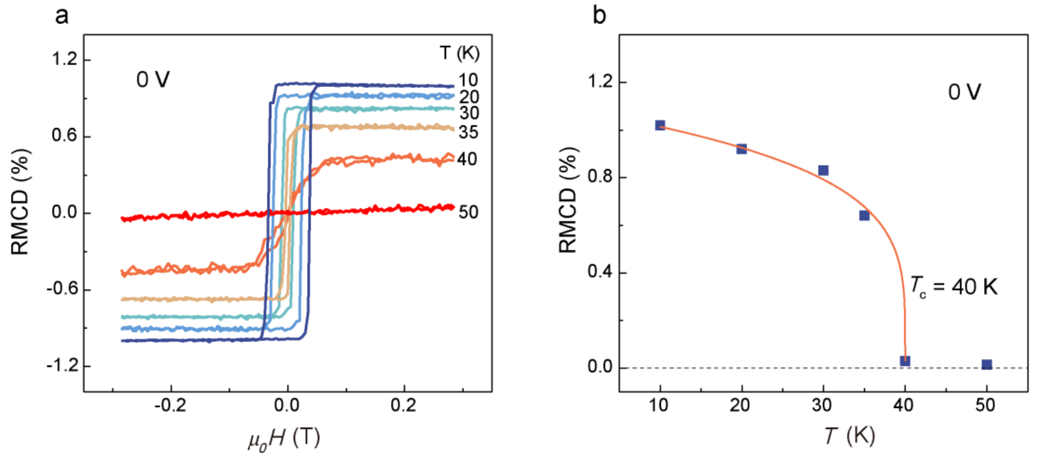

Supplementary Fig. 4 | Criticality analysis of monolayer CrI_3 at 0 V. a RMCD versus field at varying temperatures for monolayer CrI_3. The coercive field and remanent magnetization decrease as the temperature increases, eventually vanishing at 40 K. **b** Remanent RMCD signal as a function of temperature for monolayer CrI_3 at 0 V. The solid lines represent least-squares criticality fits of the form $\alpha(1 - T/T_c)^{\beta}$, while the dotted line indicates a zero RMCD signal. The Curie temperature (T_c) of monolayer CrI_3 was determined to be 40 K through fitting.

Supplementary Note 1: Raman scattering and its dependence on magnetic order in monolayer CrI_3.

We begin by presenting the RMCD loops of monolayer CrI_3 measured over a broad magnetic field range (-1.9 T to +1.9 T) at 10 K. A single jump is observed at 0.04 T, attributed to a spin-flip induced by the magnetic field. No any other phase transitions, such as those caused by interlayer antiferromagnetic coupling, are detected at higher fields, confirming that the flake is indeed a monolayer. At 50 K, above the Curie temperature ($T_c \approx 40$ K), the material exhibits paramagnetic, as confirmed by the temperature-dependent RMCD loops shown in Supplementary Fig. 4.

Raman spectra collected at 50 K in both cross- and co-linear polarization channels (denoted XY and XX, respectively) reveal three distinct peaks (Supplementary Fig. 5c). The peaks at 77.1 cm^{-1} and 127.8 cm^{-1} correspond to scattering from A_{1g} phonons, while the peak at 106.7 cm^{-1} is associated with E_g phonons. Notably, residual Raman scattering from the 127.8 cm^{-1} A_{1g} phonon is observed in the XY channel, whereas no signal from the 77.1 cm^{-1} A_{1g} phonon appears in this channel. While Raman scattering from A_{1g} phonons is typically forbidden in the XY channel, it can be activated through mechanisms such as resonance effects, enabling this observed Raman activity.

As the temperature is cooled below T_c, the monolayer CrI_3 transitions to a ferromagnetic state, as evidenced by magnetic hysteresis observed in the RMCD measurements (Supplementary Figs. 5a and 4). As shown in Supplementary Fig. 5b, there is a marked increase in the cross-linearly polarized Raman scattering from the 127.8 cm^{-1} A_{1g} phonon. However, no significant changes are observed in the Raman signals from the 77.1 cm^{-1} A_{1g} and 106.7 cm^{-1} E_g phonons in either the XX or XY channels. And the 127.8 cm^{-1} A_{1g} phonon in the XY channel serves as an effective probe for determining the Curie temperature of monolayer CrI_3. As shown in Supplementary Fig. 5d, the intensity of this phonon drops sharply around 40 K, which coincides with the Curie temperature. The in-situ temperature-dependent RMCD loops and polarization-dependent Raman scattering experiments provide complementary evidence, confirming that the CrI_3 in our device has reached the monolayer limit.

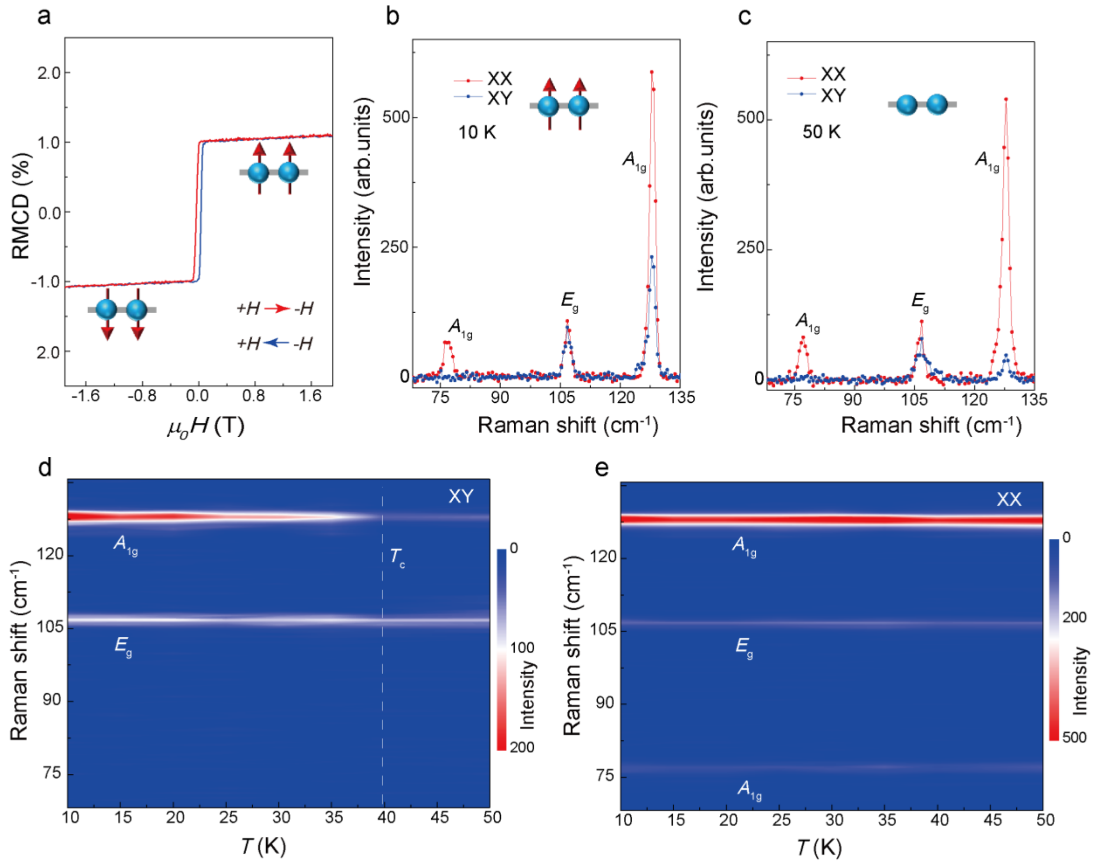

Supplementary Fig. 5 | Raman scattering and its dependence on magnetic order in 1L CrI_3. a RMCD loops of monolayer CrI_3 scanned over a high magnetic field range (±1.9 T). The RMCD curve exhibits a single step, with no additional transitions observed at higher magnetic fields, excluding interlayer antiferromagnetic coupling in multilayer CrI_3. This provides robust evidence that CrI_3 is monolayer. **b**, **c** Raman spectrum of 1L CrI_3 in the FM state at 10 K and the paramagnetic state at 50 K. Red (blue) curves correspond to co-linear (cross-linear) excitation and detection. **d**, **e** Colour maps of cross-linearly polarized (**d**) and co-linearly polarized (**e**) Raman spectra taken at a range of temperatures while warming from 10 to 50 K in a zero applied magnetic field. In cross-linearly polarized Raman spectra, Raman mode A_{1g} exhibits a marked enhancement below the phase transition temperature of 40 K due to the coupling between magnetic orders and the lattice. This transition temperature aligns with the Curie temperature determined by RMCD measurements (Supplementary Fig. 4). In contrast, the intensity of Raman modes in co-linearly polarized Raman spectra remains unchanged with temperature.

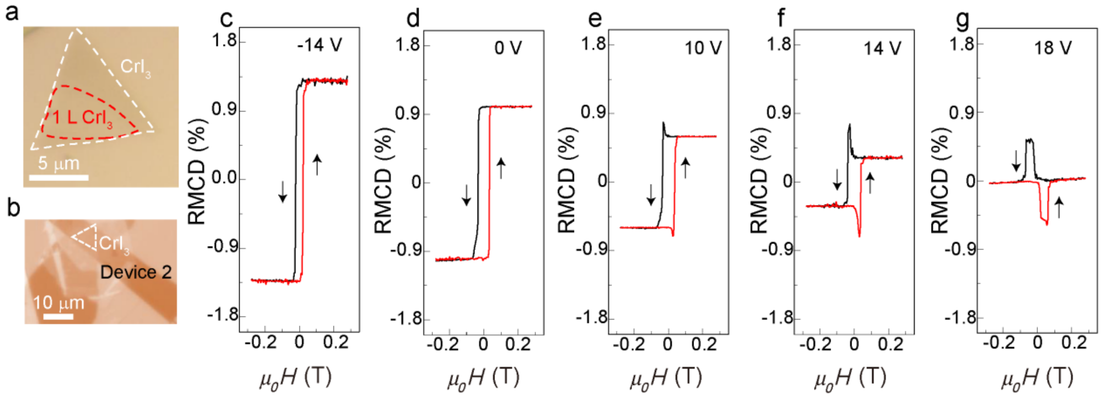

Supplementary Fig. 6 | Voltage-controlled topological circular dichroism in other monolayer CrI_3 device (device 2). a Optical microscope image of CrI_3 nanosheets exfoliated from bulk CrI_3 and adhered to the PDMS surface, and the region enclosed by the red dashed line represents the atomic-thick CrI_3 area. **b** False-color optical micrograph of the device 2, with the junction region represented by the area enclosed by a white dashed line. **c-g** RMCD versus magnetic field at different gate voltages at 10 K. The black arrows indicate the direction of the field sweep.

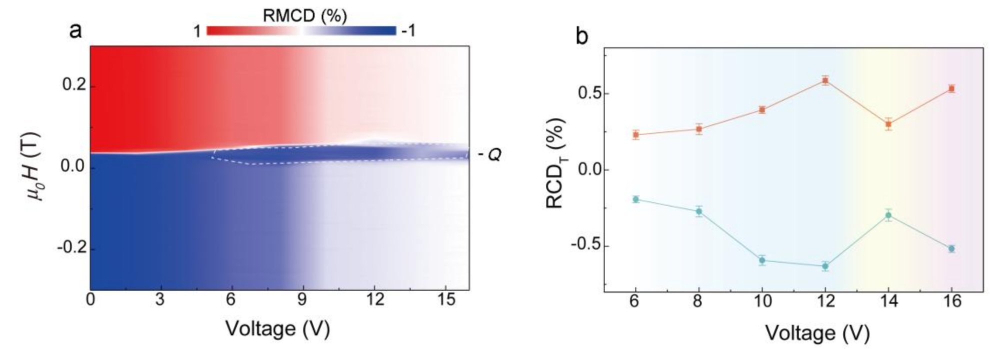

Supplementary Fig. 7 | Voltage-controlled multistage topological magnetic phase transition in monolayer CrI_3. a Intensity of the RMCD signal of a monolayer CrI_3 device as a function of both gate voltage and applied magnetic field (sweeping from negative to positive). The region bounded by white dashed lines highlights the topological magnetic phase with negative topological charge. **b** The relationship between RCD_T peak intensity and voltage, obtained through Gaussian fitting of the experimental data, is shown with error bars representing the half-peak widths.

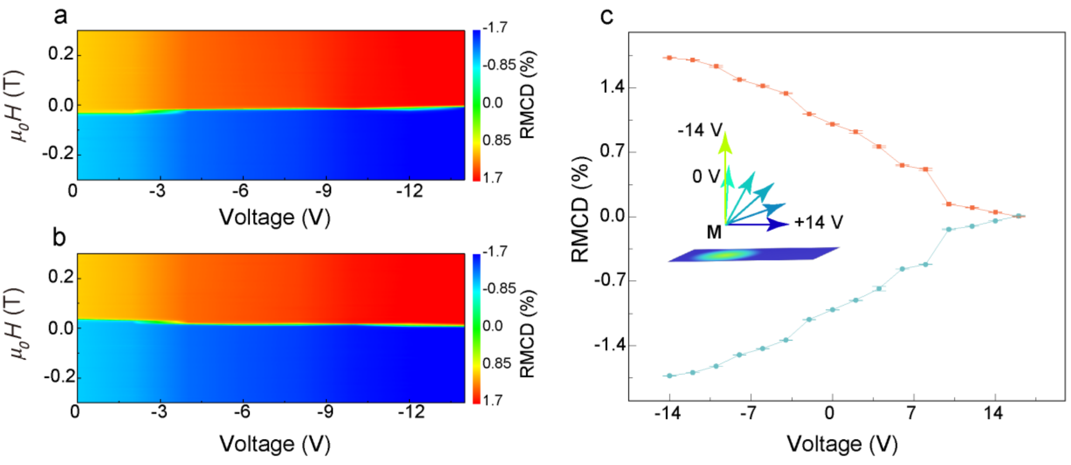

Supplementary Fig. 8 | Voltage-controlled magnetic anisotropy in monolayer CrI_3. **a** Intensity of the RMCD signal of a monolayer CrI_3 device as a function of negative voltage and applied magnetic field (sweeping from positive to negative). **b** The time-reversal process corresponding to (**a**) (sweeping from negative to positive). **c** Dependence of RMCD intensity on voltage under +0.1 T and -0.1 T magnetic fields. The error bars represent mean ± standard error of the mean. As the negative voltage increases (indicating higher hole doping concentrations), the out-of-plane magnetization are strengthened, suggesting that hole doping enhances perpendicular magnetic anisotropy. Conversely, with increasing positive voltage (indicating higher electron doping concentrations), the out-of-plane magnetization diminishes, suggesting a gradual shift from perpendicular to in-plane magnetic anisotropy (highlighted with arrows in the inset).

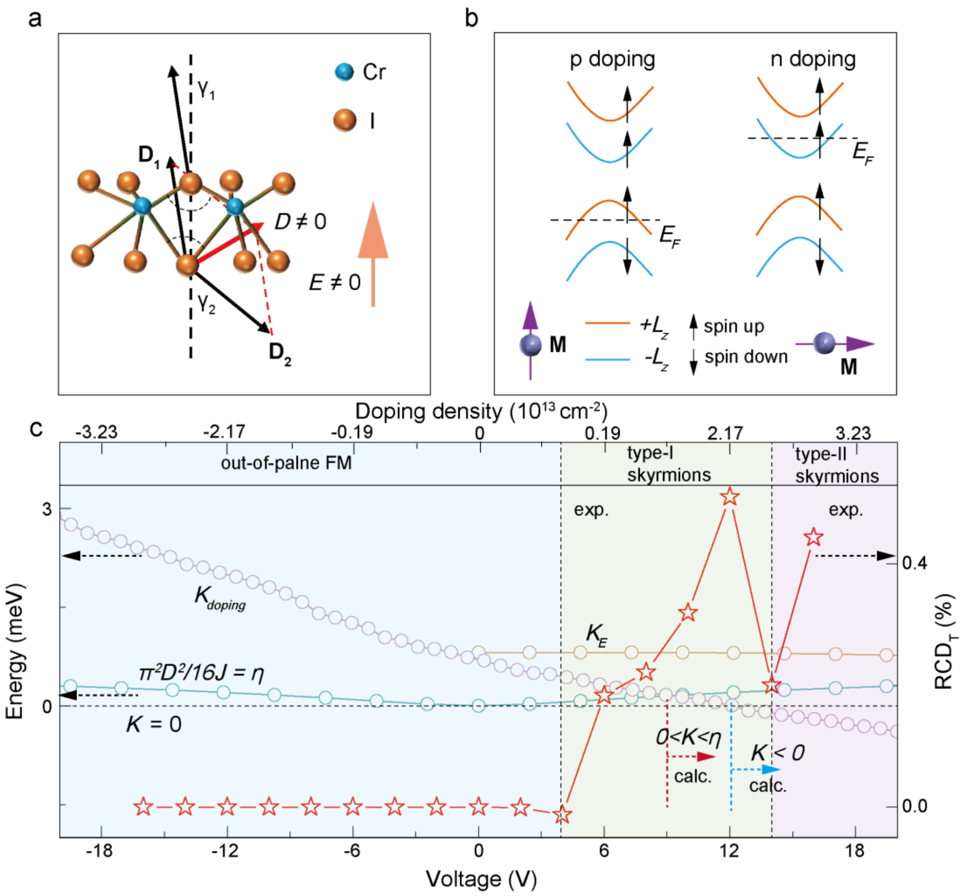

Supplementary Fig. 9 | The mechanism of voltage-controlled multistage topological magnetic phase transitions in monolayer CrI_3. **a** A schematic representation of the electric-field-induced breaking of spatial inversion symmetry in monolayer CrI_3, leading to the emergence of the DMI. **b** A schematic illustration of the band-edge electronic structure in p-doped or n-doped CrI_3, where the direction of orbital angular momentum is indicated by color coding, and spin-up and spin-down states are denoted by arrows. **c** The voltage (doping density) dependence of the factor η, the magnetic anisotropy energy (K), and the RCD_T intensity (at -0.04 T) is investigated. D is induced by the electric field, while K_{doping} is caused by electrostatic doping. Both contributions are derived from prior theoretical frameworks and quantified using interpolation methods. Notably, K_E shows negligible voltage dependence, indicating that the electric field has minimal effect on magnetic anisotropy.

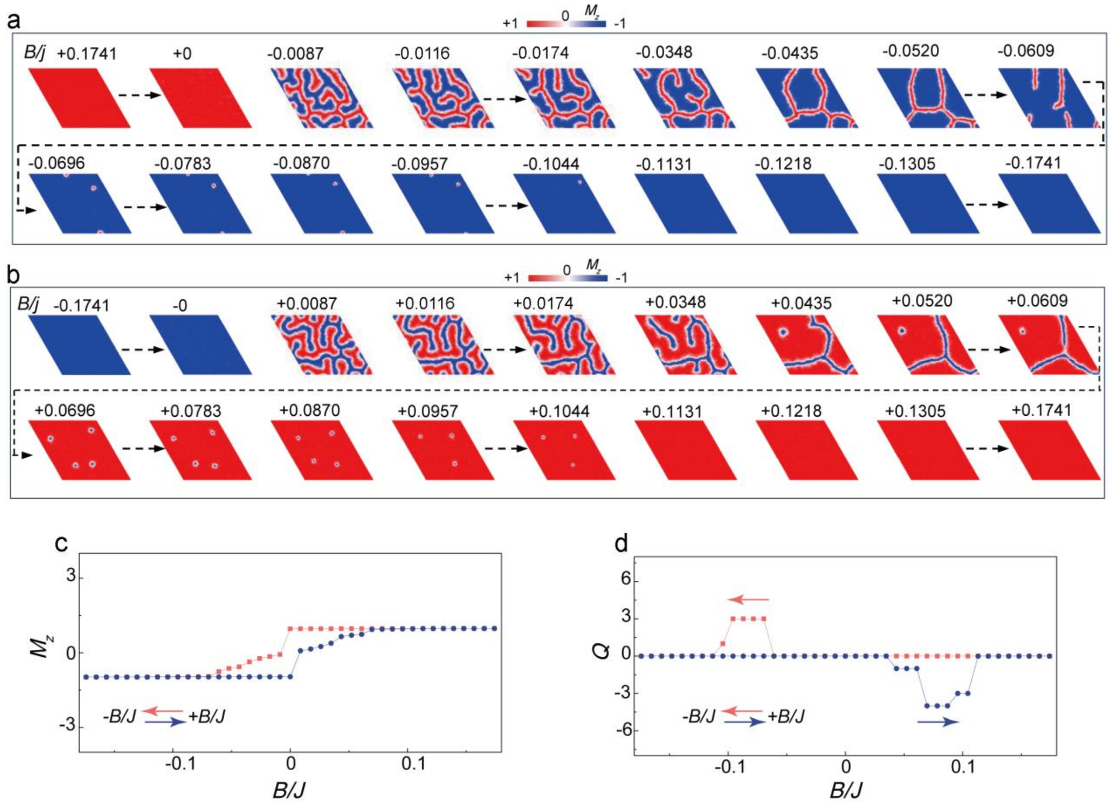

Supplementary Fig. 10 | Simulated evolution of the magnetic structures in CrI_3 induced by a magnetic field under a voltage of 8 V (perpendicular magnetic anisotropy, $K > 0$). a A series of representative magnetic domain configurations during the process of the magnetic field scanning from the positive maximum to the negative maximum. The black dashed arrow indicates the direction of the magnetic field scan. A 100×100 supercell was used for the magnetic simulation of the two-dimensional spin lattice, with colors mapping the out-of-plane magnetic moment components (M_z). **b** A series of representative magnetic domain configurations during the process of the magnetic field sweeping from the negative maximum to the positive maximum. **c** The variation of M_z with the magnetic field (M_z normalized to its saturation value). The orange line represents the magnetic field sweeping direction from positive to negative, while the blue line indicates the sweeping direction from negative to positive. **d** The evolution of the topological charge (per 10,000 spin sites) along the same hysteresis loop depicted in (**c**).

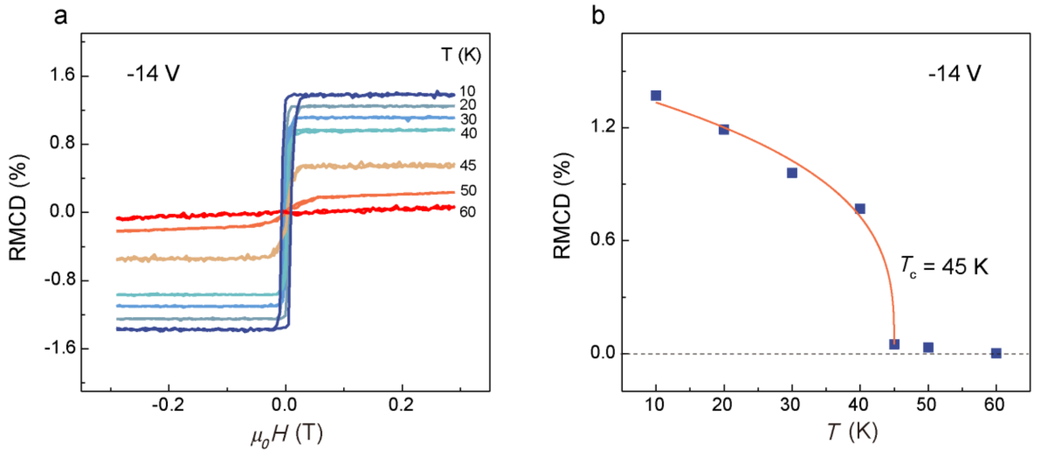

Supplementary Fig. 11 | A negative voltage increases the Curie temperature of monolayer CrI_3. a RMCD as a function of magnetic field at various temperatures for monolayer CrI_3 under -14 V. **b** Remanent RMCD signal versus temperature for monolayer CrI_3 at -14 V. Solid lines represent least-squares criticality fits of the form $\alpha(1 - T/T_c)^\beta$, while the dotted line indicates zero RMCD signal. The Curie temperature (T_c) of monolayer CrI_3 at -14 V was determined to be 45 K through fitting.

Supplementary Note 2: Theoretical simulation of the magnetic structure evolution with out-of-plane magnetic field in monolayer CrI_3 at different temperatures under 16 V bias.

Both temperature and out-of-plane magnetic field function as effective fields and play a critical role in the formation and annihilation of topological spin textures. Supplementary Fig. 12a displays the evolution of magnetic domains at a fixed temperature of $T/J = 0.0044$ as the out-of-plane magnetic field is swept from $B/J = -0.214$ to $B/J = 0.214$. The results demonstrate that skyrmions nucleate at $B/J = 0.086$ and undergo complete annihilation at $B/J = 0.197$. When the temperature is elevated to $T/J = 0.0879$, skyrmions similarly emerge at $B/J = 0.086$ but are fully annihilated at a reduced magnetic field of $B/J = 0.171$ (Supplementary Fig. 12b). The methodology used in the atomic-scale spin simulations aligns with that of the theoretical simulations performed at 16 V (Fig. 3 and Methods), with the sole distinction that temperature is incorporated as a variable. Supplementary Fig. 12c and 12d show the phase diagrams of the topological charge density in monolayer CrI_3 as functions of both temperature and magnetic field. The results reveal that the magnetic field required for the initial nucleation of skyrmions remains relatively invariant with increasing temperature (denoted by the black dashed line), whereas the annihilation field decreases systematically with rising temperature (indicated by the green dashed line). This trend is consistent with the behavior of the topological RMCD signal as a function of magnetic field and temperature observed experimentally (Fig. 4b and 4c). This consistency provides further support for the interpretation that the topological RMCD peaks originate from skyrmions.

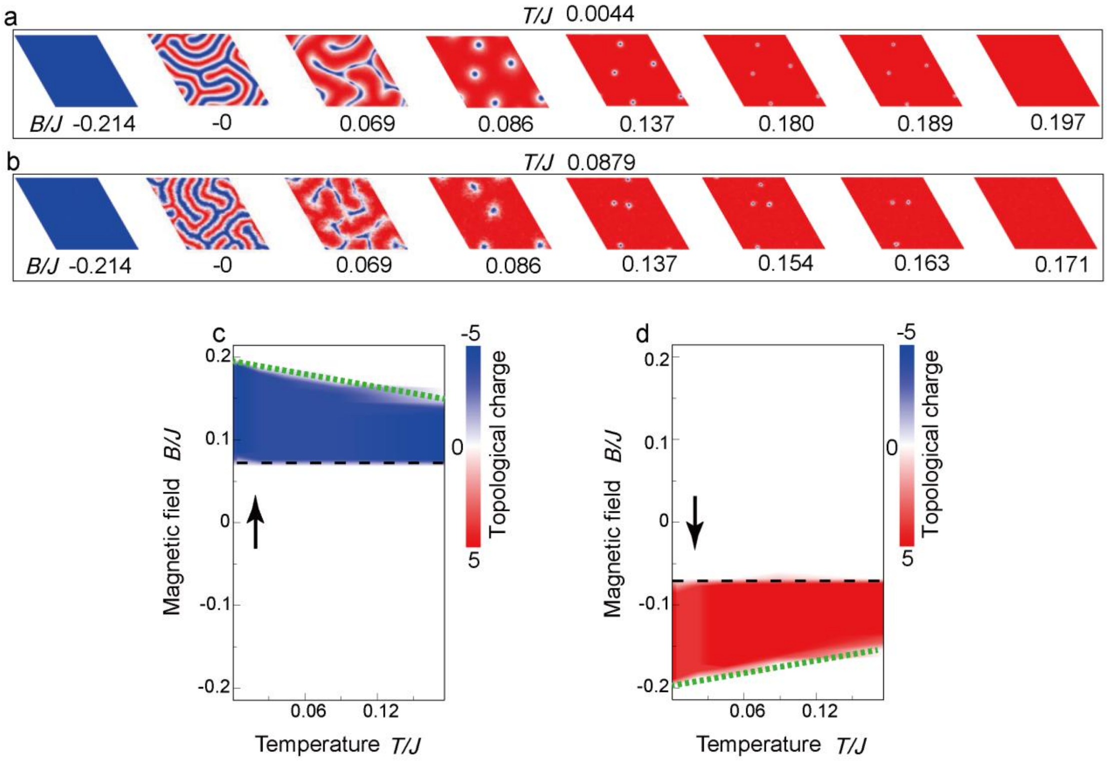

Supplementary Fig. 12 | Atomic-scale spin dynamics simulations of the magnetic structure evolution with out-of-plane magnetic field in monolayer CrI_3 at different temperatures under 16 V bias. a A series of representative magnetic domain configurations at T/J = 0.0044 during a magnetic field (B/J) sweep from negative to positive maximum. A 100×100 supercell was used for the magnetic simulation of the two-dimensional spin lattice, with colors mapping the out-of-plane magnetic moment components (M_z). **b** A series of representative magnetic domain configurations at T/J = 0.0879 during a magnetic field (B/J) sweep from negative to positive maximum. **c** The topological charge (per 10,000 spin sites) as a function of temperature and magnetic field (sweeping from negative to positive). **d** The time-reversal process corresponding to (**c**) (sweeping from positive to negative). The black arrows indicate the direction of the magnetic field sweep.

Supplementary Note 3: Temperature- and magnetic-field-dependent RCD_T induced by a 14 V gate voltage.

Supplementary Fig. 13a presents the RMCD as a function of the magnetic field at different temperatures under a 14 V gate voltage. With increasing temperature, the topological magnetic phase clearly undergoes a transition to a ferromagnetic state at approximately 25 K, followed by a subsequent transition from a weak ferromagnetism with slight perpendicular magnetic anisotropy to a paramagnetic state around 30 K. Under an applied voltage of 14 V, the T_c (30 K) of monolayer CrI_3 is lower than that of intrinsic monolayer CrI_3 (40 K, Supplementary Fig. 4), but slightly higher than the T_c (27 K) under a 16 V gate voltage. This is because the magnetic anisotropy energy decreases with increasing electron doping concentration (Fig. 3c). Supplementary Fig. 13b and 13c present the RMCD as functions of temperature and magnetic field. The results indicate that the magnetic field range for stabilizing the topological phase is narrowed as the temperature increases. Additionally, the magnetic fields corresponding to the maximum density of topological quasiparticles—the positions of the RCD_T peaks with positive topological charge (upward peaks) and negative topological charge (downward peaks)—decrease linearly with temperature, exhibiting slopes 12.4 Oe K^{-1} and 8.9 Oe K^{-1}, respectively (Supplementary Fig. 13d). Furthermore, the half-peak width of the RCD_T peaks, which characterizes the robustness of topological quasiparticles against magnetic fields, also gradually diminishes as the temperature rises. RCD_T is obtained by subtracting RCD_M from the total RMCD (Supplementary Fig. 14a), as shown in Equation (3) in the main text. The peak height and half-peak width of the RCD_T peaks are determined through Gaussian fitting of the experimental data (Supplementary Fig. 14b), following a procedure similar to that in Fig. 2c. Supplementary Fig. 13e shows the temperature dependence of RCD_T under selected magnetic fields (horizontal slices of the phase diagram in Supplementary Fig. 14a), illustrating the dependence of the critical temperature for the topological-to-ferromagnetic phase transition on the external magnetic field. The critical temperature shifts by approximately 10 K, increasing from 15 K at ±0.055 T to 25 K at ±0.025 T. By comparing the temperature dependence of RCD_T peak intensity under 14 V and 16 V voltages (Supplementary Fig. 14b), it is revealed that, at the optimal magnetic field, the density of topological quasiparticles induced under 16 V is higher compared to 14 V, but the temperature window for the existence of the topological magnetic phase is narrower.

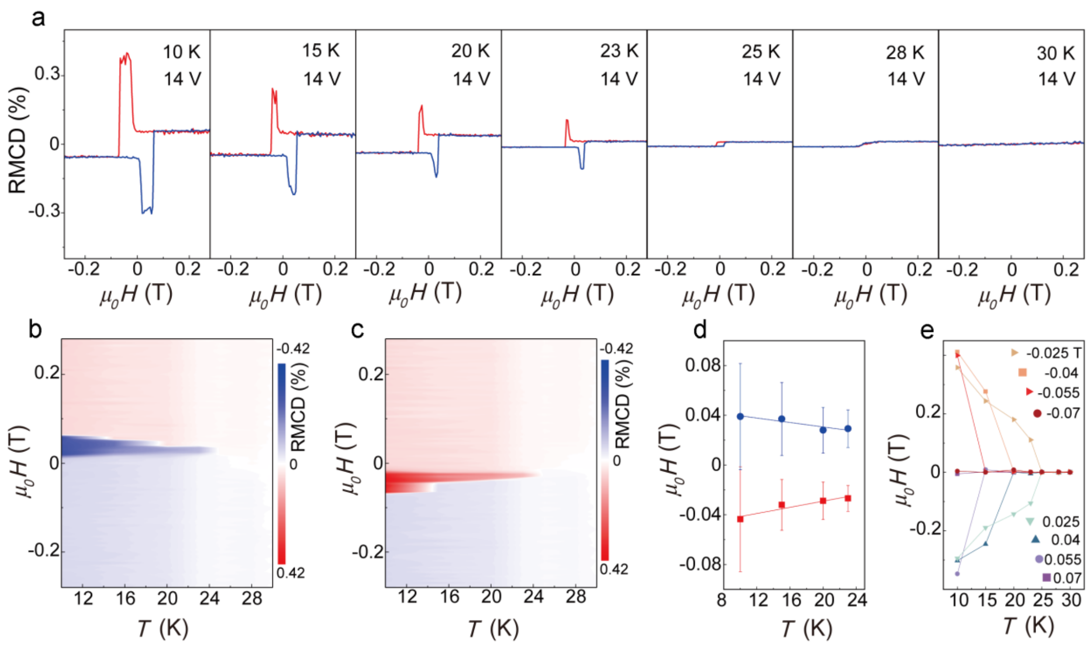

Supplementary Fig. 13 | Temperature-magnetic field phase diagram at 14 V. a Intensity of the RMCD signal of a monolayer CrI_3 device under a 14 V voltage as a function of the magnetic field at different temperatures. The orange line represents the sweeping direction from positive to negative magnetic field, while the dark blue line represents the sweeping direction from negative to positive magnetic field. **b** The RCMD intensity as a function of temperature and magnetic field (sweeping from negative to positive). **c** The time-reversal process corresponding to (**b**) (sweeping from positive to negative). **d** The RCD_T peak positions, where the density of topological quasiparticles is maximal under the corresponding magnetic fields, are plotted as a function of the temperatures. Blue solid circles and red solid squares represent the magnetic field values corresponding to the blue and red solid RMCD peaks in (**a**), respectively. Solid lines denote linear fits to the experimental data, and error bars represent the half-peak widths. The peak positions and half-peak widths are obtained through Gaussian fitting. **e** The RCD_T signal intensity as a function of temperature at various positive and negative magnetic fields indicates that the out-of-plane magnetic field significantly influences the phase transition temperature from topological magnetic to ferromagnetic.

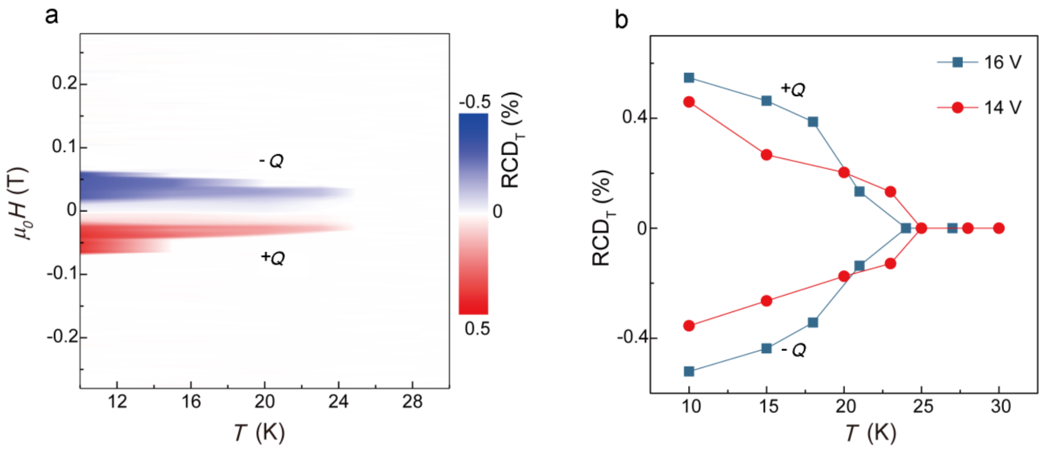

Supplementary Fig. 14 | Topological magnetic phase diagram from the RCD$_T$ as a function of temperatures and $\mu_0 H$. a Intensity of the RCD$_T$ signal of a monolayer CrI_3 device under a 14 V voltage as a function of the magnetic field and temperatures. RCD$_T$ is obtained by subtracting RCD$_M$ from the total RMCD, as described in Equation (3) and Fig. 2b. The red and blue regions represent topological magnetic phases with positive and negative topological charges, respectively. **b** The temperature dependence of RCD$_T$ peak intensities with opposite topological charges at 14 V and 16 V was determined through Gaussian fitting of the experimental data.
